# Hypomanic Experience in Young Adults Confers Vulnerability to Intrusive Imagery After Experimental Trauma: Relevance for Bipolar Disorder

**DOI:** 10.1177/2167702614527433

**Published:** 2014-11

**Authors:** Aiysha Malik, Guy M. Goodwin, Laura Hoppitt, Emily A. Holmes

**Affiliations:** 1 University of Oxford; 2 Medical Research Council Cognition & Brain Sciences Unit

**Keywords:** bipolar disorder, mental imagery, trauma film, involuntary memory, intrusive imagery

## Abstract

Emotional mental imagery occurs across anxiety disorders, yet is neglected in bipolar disorder despite high anxiety comorbidity. Furthermore, a heightened susceptibility to developing intrusive mental images of stressful events in bipolar disorder and people vulnerable to it (with hypomanic experience) has been suggested. The current study assessed, prospectively, whether significant hypomanic experience (contrasting groups scoring high vs. low on the Mood Disorder Questionnaire, MDQ) places individuals at increased risk of visual reexperiencing after experimental stress. A total of 110 young adults watched a trauma film and recorded film-related intrusive images for 6 days. Compared to the low MDQ group, the high MDQ group experienced approximately twice as many intrusive images, substantiated by convergent measures. Findings suggest hypomanic experience is associated with developing more frequent intrusive imagery of a stressor. Because mental imagery powerfully affects emotion, such imagery may contribute to bipolar mood instability and offer a cognitive treatment target.

Bipolar disorder is characterized by manic episodes (in which mood is persistently elevated) interspersed with periods of depression (*Diagnostic and Statistical Manual of Mental Disorders, 5th ed.; DSM–5*; American Psychiatric Association, 2013). It is arguably the most expensive mental health diagnosis for society (Peele, Xu, & Kupfer, 2003) and the seventh leading global cause of years lost to disability (World Health Organization, 2008). Pharmacological and psychological treatments are as yet inadequate for the majority of patients (Geddes & Miklowitz, 2013). Patients with bipolar disorder often have ongoing mood instability—that is, highly variable week to week interepisodic symptom ratings of both mood elevation (hypomania) and depression (Bonsall, Wallace-Hadrill, Geddes, Goodwin, & Holmes, 2012; Bopp, Miklowitz, Goodwin, Rendell, & Geddes, 2010; Gruber, 2011; Henry et al., 2008; Strejilevich et al., 2013). In addition to hypomanic and depressed mood, anxiety is common (Akiskal et al., 2006; Merikangas et al., 2007). Anxiety reactions to stressful events remain to be better understood in the context of bipolar disorder.

We have previously proposed that mental imagery amplifies emotional reactions to events in bipolar disorder, contributing to bipolar mood instability (Holmes, Geddes, Colom, & Goodwin, 2008). Specifically, we hypothesized that bipolar disorder is associated with a heightened susceptibility to developing intrusive mental imagery, such as visual reexperiencing after stressful (or other highly emotional) experiences. This hypothesis extends to those vulnerable to bipolar disorder (e.g., those with hypomanic experience).

Mental imagery involves “seeing with the mind’s eye” and “hearing with the mind’s ear” (Kosslyn, Ganis, & Thompson, 2001). Compared to verbal thought, imagery has a more powerful impact on emotion (Holmes, Mathews, Mackintosh, & Dalgleish, 2008). Distressing, intrusive mental imagery has been noted in a number of psychological disorders, particularly anxiety (Brewin, Gregory, Lipton, & Burgess, 2010; Holmes & Mathews, 2010). Examples include flashbacks in posttraumatic stress disorder (PTSD; Ehlers, Hackmann, & Michael, 2004; Grey & Holmes, 2008; Rubin, Boals, & Bernsten, 2008) and negative-self imagery in social phobia (Hackmann, Clark, & McManus, 2000). However, despite the high comorbidity of anxiety in bipolar disorder (up to 90%; Merikangas et al., 2007), research on mental imagery within bipolar disorder is scarce.

In a cross-sectional study of patients with bipolar disorder divided according to their mood stability over the past months, higher levels of intrusive, prospective mental imagery were associated with greater mood instability (Holmes et al., 2011). Patients scored higher than did nonclinical controls on this measure, as well as on spontaneous use of imagery (Holmes et al., 2011). Bipolar disorder, compared to nonclinical controls, has been associated with superior visual mood-dependent memory (Nutt & Lam, 2011). Compared to patients with unipolar depression, in those with bipolar depression imagery associated with suicidal ideation (flash-forwards) was more preoccupying and compelling (Hales, Deeprose, Goodwin, & Holmes, 2011).

Individuals with hypomanic experience (a less severe form of mania) are argued to have a “bipolar phenotype” (Rock, Chandler, Harmer, Rogers, & Goodwin, 2013). Hypomanic experience has also been associated with higher levels of intrusive future imagery (Deeprose, Malik, & Holmes, 2011) as well as more general intrusive imagery (McCarthy-Jones, Knowles, & Rowse, 2012). Risk for mania, again in a nonclinical sample, predicted imagery in the form of daydreaming (Meyer, Finucane, & Jordan, 2011). However, such studies are cross-sectional and cannot inform whether there is a causal link between hypomanic experience and visual reexperiencing after a stressful event.

The current study used a prospective experimental design. Participants assessed for hypomanic experience experienced a controlled laboratory stressor—a film of traumatic events. The trauma film paradigm has been long used (Lazarus, 1964) in stress and anxiety research (for a review, see Holmes & Bourne, 2008). Recent studies have included the impact of intrusive memories of the film following alcohol use or analogue therapy techniques (e.g., Bisby, Brewin, Leitz, & Curran, 2009; Hagenaars & Arntz, 2012; Verwoerd, Wessel, de Jong, Nieuwenhuis, & Huntjens, 2011).

We hypothesized that those participants with significant hypomanic experience when compared to a control group (with lower levels of hypomanic experience) would experience a greater number of intrusive mental images of the stressful event (trauma film). It is perhaps counterintuitive to predict that after a stressor those with more “positive” hypomanic tendencies (such as being self-confident, hyper, talkative) will be more vulnerable to visual reexperiencing of the stressor. However, it is important to ascertain this as it would mean that stressful events could have a greater impact on this population, at least (and perhaps especially) in an imagery form.

## Method

### Participants and procedure

A total of 110 participants (54 men and 56 women) provided their written informed consent (Central University Research Ethics Committee, University of Oxford) and completed the study. Participants described their ethnicity as White British (54%), Other White background (26%), Chinese (5%), Asian (5%), Mixed White and Asian (5%), Mixed White and Black Caribbean (2%), or Other background (3%). Participants were recruited via poster and online advertisements in the local area and at universities, seeking those with and without hypomanic experience. Participants who were eligible (and provided consent) attended two laboratory sessions separated by 6 days. The Mood Disorder Questionnaire (MDQ; Hirschfeld et al., 2000) and the Eysenck Personality Questionnaire–Neuroticism short scale (EPQN; Eysenck, Eysenck, & Barrett, 1985) were given at screening. The MDQ was used to divide participants according to hypomanic experience. The MDQ is a self-report screening tool that assesses lifetime experiences of (hypo)mania. In all, 50 participants were categorized into the high MDQ group (scoring ≥ 7; range = 7–15) and 60 into the low MDQ group (scoring ≤ 6; range = 0–6). In Session 1, psychiatric screening and baseline measures (including measures of emotion, general imagery ability, and previous trauma history) were completed. Following baseline measures, participants watched the stressful film and completed the associated ratings.

For the next 6 days participants (in response to prompts sent via mobile phone) reported any intrusive images of the film. At the follow-up session in the laboratory (Day 6), participants completed convergent measures of intrusive imagery, the Intrusion Provocation Task and the Impact of Event Scale–Revised (IES-R; Weiss & Marmer, 1997), as well as measures of recognition memory for the film, behavior change associated with the film, and compliance at responding to mobile phone prompts. Participants were included if they did not have current (hypo)mania and depression (Mini International Neuropsychiatric Interview–Plus; Sheehan et al., 1998), had use of a mobile phone (one individual did not), and were able to attend two sessions. Table 1 summarizes baseline characteristics for both groups.

**Table 1.** Demographic Characteristics, Emotional Measures, Trauma History, and General Imagery Measures for High MDQ and Low MDQ Groups

	High MDQ (*n* = 50)		Low MDQ (*n* = 60)		Analysis			
Characteristic/measure	*M*	*SD*	*M*	*SD*	*t*	*df*	*p*	*d*
Age (years)	25.48	10.11	28.02	9.66	1.34	108	.18	0.26
Emotional measures								
MDQ	10.62	2.03	2.45	2.05	20.89	108	<.001	4.02
Beck Depression Inventory–Second Edition (BDI-II)	8.60	6.93	3.00	3.66	5.42	108	<.001	1.04
Eysenck Personality Questionnaire–Neuroticism (EPQN)	5.58	2.49	2.40	2.23	7.05	108	<.001	1.36
Traumatic Experiences Questionnaire (TEQ)	1.58	1.34	1.22	1.32	1.43	108	.16	0.28
General imagery measures								
Spontaneous Use of Imagery Scale (SUIS)	39.36	6.72	32.50	9.03	4.44	108	<.001	0.85
Impact of Future Events Scale (IFES)	30.78	16.46	18.63	10.58	4.68	108	<.001	0.90

Note: MDQ = Mood Disorder Questionnaire.

### Measures

#### Psychiatric screening

The Mini International Neuropsychiatric Interview–Plus (MINI-Plus; Sheehan et al., 1998) was used as a brief structured interview to assess current and lifetime major Axis I disorders in the *Diagnostic and Statistical Manual of Mental Disorders, 4th ed., Text Revision* (*DSM–IV–TR*; American Psychiatric Association, 2000) and *International Statistical Classification of Diseases and Related Health Problems 10th Revision* (World Health Organization, 1992). The MINI has been applied in a variety of settings and shows good concordance with other structured clinical interviews, excellent interrater reliability (majority of kappa values .90 or higher), and very good retest reliability, with 61% of kappa values greater than .75 (Sheehan et al., 1998).

#### Emotional measures

The MDQ (Hirschfeld et al., 2000) assessed hypomanic experience and was used to distinguish groups with high versus low hypomanic experience (high MDQ vs. low MDQ). It asks individuals about periods in their life when they were not their usual self and, for example, had more energy, more confidence, or more irritability than usual. The MDQ is a self-report measure comprising three primary sections. The first section contains 13 yes/no items indexing manic/hypomanic symptoms (e.g., “Has there ever been a period of time when you were not your usual self and you had much more energy than usual?”). The second section addresses the co-occurrence of symptoms by asking (with a yes/no answer) whether or not several of the symptoms from the first section ever happened during the same period. The third section requires individuals to report the severity of the symptoms on a 4-point scale: *no problem, minor problem, moderate problem*, or *serious problem*. Higher scores reflect greater hypomanic experience, and in line with previous literature (Calabrese et al., 2006) the cutoff score of 7 was used to divide high versus low groups in the current study. The MDQ has high levels of internal consistency (α = .90).

The MDQ has been validated by looking at the rate of bipolar diagnoses using *DSM–IV–TR* (American Psychiatric Association, 2000) criteria at interview. The positive predictive validity of a score of 7 on items in Section 1 (without positive responses to the questions in Section 2, co-occurrence, or Section 3, severity of symptoms) is 34%; a score of 7 or more (plus positive responses to Sections 2 and 3) is 62%; a score of 0 is 0%. The present split was made at 7 or more (high MDQ), and members of this group are a higher risk group for a bipolar diagnosis (Rock et al., 2013). Furthermore, we note that the MDQ has a sensitivity of 87% and a specificity of 61% in a patient sample (Calabrese et al., 2006). In the general population the MDQ has a sensitivity of 57% and a specificity of 82% (Hirschfeld et al., 2003).

The Beck Depression Inventory–Second Edition (BDI-II; Beck, Steer, & Brown, 1996) is a 21-item self-report measure of depressive symptomatology. Participants rate items according to which best describes their mood in the preceding 2 weeks. The BDI-II has high internal consistency in clinical outpatients (α = .92) and student samples (α = .93; Beck et al., 1996).

The EPQN (Eysenck et al., 1985) contains 12 items requiring a yes or no response. The sum of yes responses was used to measure a general neuroticism tendency, with higher scores indicating greater neuroticism. The EPQN shows good internal consistency (α = .84 and .80 for males and females, respectively; Eysenck et al., 1985).

#### Trauma history measure

The Traumatic Experiences Questionnaire (TEQ) is a 12-item checklist assessing trauma history based on the *DSM–IV–TR* (American Psychiatric Association, 2000) PTSD Criterion A items of the Posttraumatic Diagnostic Scale (Foa, 1995).

#### General imagery measures

Two self-report scales served to measure general mental imagery use and experience (Pearson, Deeprose, Wallace-Hadrill, Burnett Heyes, & Holmes, 2013). The Spontaneous Use of Imagery Scale (SUIS; Reisberg, Pearson, & Kosslyn, 2003) is a measure of tendency to use imagery. It has 12 items that are each a statement related to spontaneous use of imagery (e.g., “When I hear a radio announcer or DJ I’ve never actually seen, I usually find myself picturing what they might look like”). Each item is rated on a 5-point scale in terms of the appropriateness of that statement. Scores range from a minimum of 12 (indicating *no use*) to 60 (*high use*; Reisberg et al., 2003). The SUIS has an internal consistency of α = .98 (Reisberg et al., 2003).

The Impact of Future Events Scale (IFES; Deeprose & Holmes, 2010) was designed to assess response to intrusive imagery of the future and was based on the Impact of Event Scale–Revised (Weiss & Marmer, 1997). Participants are asked to identify three future events that they had been imagining over the past 7 days and state whether each event was positive or negative. They are then asked to respond to 24 items that assess intrusive preexperiencing, avoidance, and hyper-arousal relating to imagery of their chosen future events. For example, items include “Pictures about the future popped into my mind” (intrusive preexperiencing), “I stayed away from reminders of the future” (avoidance), and “I was jumpy and easily startled” (hyperarousal). The IFES has been shown to have acceptable test–retest reliability (.73) and good internal consistency (α = .87; Deeprose et al., 2011).

#### Stressful film

We exposed participants to a film with traumatic content consistent with *DSM–5* Criterion A for psychological trauma—actual or threatened death and serious injury (*DSM–5*; American Psychiatric Association, 2013). The film lasted 17 min 48 s (played using Windows Media Player on a 17-inch visual display unit monitor in 32-bit color) and consisted of 11 scenes of traumatic content taken from films used in previous studies (Holmes, James, Kilford, & Deeprose, 2010; Lang, Moulds, & Holmes, 2009). It included scenes of road traffic accidents, graphic surgery, and suicide. Participants rated their level of fear, anxiety, sadness, and depression pre- and postfilm on 100 mm visual analogue scales (VASs) anchored from *not at all* to *extremely*. The four ratings were combined to create mean mood prefilm and mean mood postfilm scores. Following film viewing, participants were asked, “Please indicate how much attention you paid to the film you had just seen,” using a rating scale anchored from 0 (*none at all*) to 10 (*total attention*), and “Please indicate how personally relevant you found the film,” on a rating scale anchored from 0 (*not at all*) to 10 (*extremely*). Intrusive imagery related to this film footage was then tracked over the next 6 days (see the next section).

#### Film-related intrusive imagery measures

##### Intrusive imagery monitoring via mobile phone—SMS

Participants were sent three daily mobile phone SMS (short messaging service) prompts for 6 days (morning, afternoon, evening) after the first laboratory session to monitor any intrusive images of the film. An intrusive image (or flashback) was defined as a mental image (not a purely verbal thought) from the stressful film that “pops into your mind spontaneously, out of the blue, without you deliberately thinking about it” and could be fleeting or vivid. Participants were asked to only report intrusive images from the film itself (i.e., not of other events) and verify these by describing the content in an SMS reply to their SMS prompt. Each SMS prompt gave participants instructions on how to respond, followed by an example response: “Please respond with the number of flashbacks of the film, the vividness rating and the image descriptions in the next 15 minutes. Thanks! e.g. ‘I4 V7 I saw an old classroom.’” Thus, participants replied by SMS (i.e., by text message) with the number of intrusive images they had experienced since the previous message sent to them (including zero), a vividness rating from 1 (*no image at all*) to 7 (*as clear as normal vision*), and a text-based description of the content of any intrusive images experienced. All SMS prompts were sent from a central mobile phone connected to a secure desktop computer, and the raw data were automatically stored in Microsoft Excel. Participants were given credit-card-size reminder instructions to keep in their wallet of how to respond to the SMS. All SMS intrusive image descriptions were assessed for whether or not they matched in content to the film (Table 2, Percentage Match). Only those intrusive images that matched the film were included in the analyses. The total number of intrusive images (the main outcome variable) was calculated for each participant.

**Table 2.** Scores for High and Low MDQ Groups in Terms of Response to the Film, Response to Mobile Phone Prompts, Frequency of Intrusive Images (in Daily Life via SMS and in a Laboratory Task), Impact of Event Scale Score, Verbal Recognition Memory for the Film, and Behavior Change

	High MDQ (*n* = 50)		Low MDQ (*n* = 60)		Analysis			
Variable	*M*	*SD*	*M*	*SD*	*t*	*df*	*p*	*d*
Mood prefilm	5.66	6.33	4.27	5.67^a^				
Mood postfilm	10.85	8.88	8.30	7.01				
Attention paid to film	9.24	0.74	9.42	0.65	1.33	108	.19	0.26
Personal relevance of film	3.86	2.36	3.35	2.52	1.09	108	.28	0.21
Percentage of SMS prompts responded to	70.20	21.57	81.57	13.70	3.35	108	.001	0.64
Match of intrusive imagery content (via SMS) with film content (%)	87.13	29.04	89.24	27.00	0.40	108	.69	0.08
Frequency of SMS-prompted intrusive images	11.02	10.61	5.78	6.90	3.11	108	.002	0.60
Vividness of SMS-prompted intrusive images	3.75	1.39	3.38	1.78	1.18	108	.24	0.23
Frequency of intrusive images triggered by Intrusion Provocation Task (IPT)	10.26	9.51	5.27	5.29	3.48	108	.001	0.67
Impact of Event Scale–Revised Total Score (IES-R)	0.67	0.59	0.38	0.34	3.22	108	.002	0.62
Verbal Recognition Memory Test: Percentage correct	62.73	11.32	62.05	9.93	0.34	108	.74	0.07
Behavior Change Questionnaire	13.06	13.34	7.04	8.91	2.82	108	.006	0.54

Note: MDQ = Mood Disorder Questionnaire; SMS = short message service via mobile phone.

a See the results section for repeated measures ANOVA.

##### Intrusion Provocation Task

The Intrusion Provocation Task (IPT) is a laboratory-based task (Lang et al., 2009) providing a standardized trigger for film-related intrusive images. Participants viewed 11 still pictures taken from the film for 2 s each on Microsoft PowerPoint (2003). Each picture was of a scene preceding one of the most distressing parts of the film (i.e., those scenes known to provoke intrusive imagery). After viewing all pictures, participants were instructed to close their eyes for 2 min while placing a finger on a keyboard. Participants recorded the occurrence of any intrusive images by tapping a designated key each time they experienced an intrusive image of the film. After the 2 min participants described aloud the content of each intrusive image so that they could be verified as from the film.

##### Impact of Event Scale–Revised

The IES-R (Weiss & Marmer, 1997) is a widely used 22-item self-report clinical measure of posttraumatic stress symptoms. The IES-R has three subscales (measuring reexperiencing, avoidance, and hyperarousal) that show a high level of intercorrelation (*r* values between .52 and .87; Creamer, Bell, & Failla, 2003). The total score on the IES-R, which shows good internal consistency (α = .96; Creamer et al., 2003), was used as a convergent measure of general trauma-related symptomatology, as anchored to the film, in the 6 days following film viewing.

#### Recognition memory for the film

The Verbal Recognition Memory Test was used (Holmes et al., 2010). Participants rated 22 statements as true or false in terms of whether or not they were related to the previously seen film. Each film scene had two statements related to it, one a true scene from the film and one a false scene from the film. For example, from a film scene depicting a car crash, the two statements were “The man with the shot-gun wore a blue jumper” and “Only one policeman was on the scene.”

#### Behavior change

The Behavior Change Questionnaire, bespoke for this study, assessed how much the stressful film had affected related safety and avoidance behaviors. Participants respond to seven items (e.g., “I have been more aware and take greater care when crossing the road” and “I have been more aware of wearing a seatbelt”) on a 100 mm scale from *not at all* to *very much so.*

### Data analytic plan

Independent samples *t* tests with group (high vs. low MDQ) as the between-subjects factor were used to compare groups. The exception to this was for the categorical variables, for which a chi-square test was used. A repeated measures analysis of variance (ANOVA) was used to compare prefilm and postfilm mood change between groups. All analyses were conducted with alpha set at .05 and were performed using SPSS Version 17.0 for Windows.

## Results

### Baseline characteristics

The two groups (high vs. low scores on the MDQ) were not significantly different in terms of gender, χ^2^(1) = 0.95, *p* = .33, Cramer’s *V* = .09, ethnicity (White British vs. other groups combined), χ^2^(1) = 3.42, *p* = .06, Cramer’s *V* = .18, and whether or not they were currently in education, χ^2^(1) = 0.03, *p* = .86, Cramer’s *V* = .02. Furthermore the two groups did not differ in terms of age or previous trauma history (see Table 1). Compared to the low MDQ group, the high MDQ group scored significantly higher on all emotional measures (Table 1). The high MDQ group also scored higher for general mental imagery ability (SUIS) and experience of intrusive future imagery (IFES; Table 1).

Analyses of the prevalence of lifetime and current Axis I disorders, as ascertained by the MINI-Plus, indicate that compared to the low MDQ group, the high MDQ group reported a higher prevalence of lifetime mood disorders, χ^2^(1) = 15.89, *p* < .001, Cramer’s *V* = .38 (high MDQ *n* = 24, low MDQ *n* = 8), current anxiety disorders, χ^2^(1) = 7.10, *p* = .008, Cramer’s *V* = .25 (high MDQ *n* = 11, low MDQ *n* = 3), and lifetime and current substance use with impairment: lifetime χ^2^(1) = 8.80, *p* = .003, Cramer’s *V* = .28 (high MDQ *n* = 9, low MDQ *n* = 1), current, χ^2^(1) = 10.35, *p* = .001, Cramer’s *V* = .31 (high MDQ *n* = 8, low MDQ *n* = 0).

### Pre- and postfilm mood and manipulation checks

Viewing the stressful film resulted in a predicted significant increase in negative mood from pre- to postfilm, *F*(1, 108) = 63.94, *p* < .001, η_p_^2^ = .37. This immediate impact of film viewing was not significantly different between groups, *F*(1, 108) = 2.64, *p* = .11, η_p_^2^ = .02, nor was there a significant time by group interaction, *F*(1, 108) = 1.02, *p* = .32, η_p_^2^ = .009 (Table 2). High and low MDQ groups did not significantly differ in attention paid to the film or in how personally relevant the film was (Table 2).

### Adherence to intrusive image monitoring via mobile phone

There was a high match between the content of the intrusive image descriptions participants sent via mobile phone SMS and scenes in the film viewed (*M* = 88.28%, *SD* = 27.83; Table 2). Two examples of intrusive images reported via SMS include “Saw the girl who got her legs trapped between her dead boyfriend and the wall she was sat on (car crash)” and “I saw a man bleeding whilst shaving.” The high MDQ group responded to significantly fewer SMS intrusive imagery prompts than did the low MDQ group (Table 2).

### Number of intrusive images of the stressful film

**Fig. 1.**
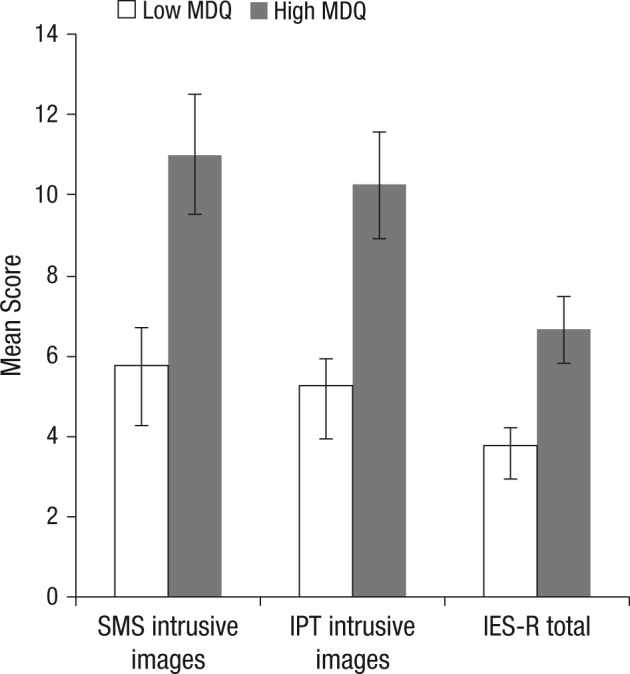
Mean frequency of film-related intrusive images reported via short messaging service (SMS) prompts, intrusive images triggered by the Intrusion Provocation Task (IPT), and Impact of Event Scale–Revised Total Score (IES-R) for high Mood Disorder Questionnaire (MDQ; *n* = 50) and low MDQ groups (*n* = 60). The IES-R is multiplied by 10 to fit the graph scale. Error bars represent ±1 standard error of the mean.

Key to predictions, the high MDQ group had significantly more frequent intrusive images than the low MDQ group, as reported via SMS over the 6 days, with almost double the mean number (Table 2 and Fig. 1).

Convergent measures revealed that compared to the low MDQ group, the high MDQ group reported significantly more intrusive images when given a trigger in the laboratory at the 6-day follow-up (IPT; Table 2 and Fig. 1). Furthermore, the high MDQ group also scored significantly higher on the clinical questionnaire measure of posttraumatic stress symptoms as related to the film (IES-R; Table 2 and Fig. 1).

The high MDQ group indicated that the film had a significantly greater effect on changing their behavior than the low MDQ group in the 6 days following viewing the stressful film (Table 2). Scores on the Verbal Recognition Memory Test confirmed no group difference in recognition memory for the film (Table 2).

### Sensitivity analysis

Results were recalculated after removing all cases with current anxiety diagnoses (i.e., 11 from the high MDQ group and 3 from the low MDQ group). The main findings in Table 2 remained the same (i.e., all statistically significant differences remained).

## Discussion

The findings present prospective evidence that those with significant hypomanic experience are vulnerable to visual reexperiencing (intrusive imagery) after a stressful event. Individuals scoring high versus low for hypomanic experience were exposed to a controlled laboratory stressor (a film of traumatic events). The impact of film viewing on subsequent intrusive images to that film was assessed for 6 days via mobile phone in daily life. In line with the key prediction, the high MDQ group reported approximately twice as many intrusive images than the low MDQ group. Findings in daily life were supported by convergent results on a laboratory based task (IPT; designed to trigger intrusions using stills from the film) and a clinical questionnaire for posttraumatic symptomatology (IES-R). Compared to the low MDQ group, the high MDQ group reported increased safety and avoidance behaviors linked to film content. Replicating previous findings, the high MDQ group had higher scores of general imagery use and prospective imagery (Deeprose et al., 2011). The difference between groups in intrusive image frequency was unlikely due to underlying recognition memory of the film because scores on the Verbal Recognition Memory Test were comparable. Furthermore, groups differed on neither emotional impact of film viewing nor its personal relevance.

We selected young adults with significant hypomanic experience, rather than patients with bipolar disorder. This allowed us to avoid confounds relating to chronic depressive symptoms, the use of multiple psychotropic medications, and multiple severe episodes of illness. A central assumption is that the high MDQ group members display psychopathology relevant to bipolar disorder–hypomania. The experience of different degrees of elation defines the bipolar phenotype (Rock et al., 2013). Furthermore, our high MDQ group already presented with a greater proportion of lifetime mood disorders, current anxiety disorders, and substance use with impairment (plus increased levels of baseline emotional measures). This parallels comorbidities reported in bipolar patient samples (Merikangas et al., 2011) despite our exclusion of current (hypo)mania or depressed mood.

The link between MDQ group and current anxiety is consistent with increasing interest in anxiety within bipolar disorder (Akiskal et al., 2006; Dickstein et al., 2005; Merikangas et al., 2007; Otto et al., 2006). *DSM–5* has introduced the bipolar specifier “with anxious distress” (American Psychiatric Association, 2013, p. 146). In the current experiment, a sensitivity analysis showed that the main findings were unchanged after removal of individuals screening positive for current anxiety disorders, indicating this difference was unlikely to be the driver. Critically however, if, as our findings suggest, stressful, anxiety-provoking events are twice as likely to trigger intrusive imagery in individuals with hypomanic experience, then such individuals may be particularly vulnerable to anxiety-provoking situations.

The limitations of this study include, first, the validity of a film with traumatic content as a model for a stressor and intrusive image development (see also Bourne, Mackay, & Holmes, 2013; Holmes, James, Coode-Bate, & Deeprose, 2009). Film content was consistent with *DSM–5* criteria for psychological trauma—exposure to actual or threatened death and serious injury (American Psychiatric Association, 2013, p. 146). A change since *DSM–IV–TR* (American Psychiatric Association, 2000) is that *DSM–5* allows for exposure through electronic media, television, movies, or pictures, at least if in work-related settings (American Psychiatric Association, 2013). In some situations (e.g., police viewing a murder via CCTV) films may produce actual PTSD symptoms. Overall, a trauma film can provide a useful experimental analogue for a stressful event. A second limitation is that the high MDQ group responded to fewer SMS prompts compared to the controls. However, despite poorer compliance, the high MDQ group reported significantly more intrusive images in daily life, and this was supported by laboratory-based convergent measures. Finally, although verbal thoughts versus images after trauma films have been shown to be different phenomena (Hagenaars, Brewin, van Minnen, Holmes, & Hoogduin, 2010), we do not have data on this in the current study.

What might be the implications of these findings in relation to other psychopathologies? As imagery can be considered a transdiagnostic process (Holmes & Mathews, 2010) predictions about intrusive imagery in the current experiment need not be limited to a sample with hypomanic experience (or bipolar disorder). Following a provoking event other disorders featuring problematic mental imagery (such as social phobia, Hackmann et al., 2000; or psychosis, Morrison et al., 2002) may also be predicted to display elevated rates of reexperiencing. In contrast those disorders in which verbal rumination dominates, such as unipolar depression (Fresco, Frankel, Mennin, Turk, & Heimberg, 2002) may display a lowered propensity for some forms of emotional mental imagery (Holmes, Geddes, et al., 2008). Finally, as hypomanic experience is associated with elated mood states, it is possible that what is unique to our sample (compared to depression, anxiety disorders, or psychosis) might be an increased likelihood for intrusive visual reexperiencing following both a highly stressful and a highly “positive” elation-related event.

Such predictions suggest various research avenues. First, as outlined previously, it is likely that across a range of disorders stressful events will lead to intrusive reexperiencing. Future work using the trauma film paradigm should compare responses of individuals with hypomanic experience versus other traits such as depressed mood, social anxiety, and schizotypy, using a between-groups design. Studies to date have been limited to single traits and correlational designs (Holmes & Steel, 2004; Laposa & Alden, 2008). Do findings extend to individuals with clinical levels of distress? Future studies could prospectively test the prediction that people with bipolar disorder will be more prone to intrusive reexperiencing in response to naturalistic traumatic events (such as disasters) versus control groups.

Second, this study has focused on intrusive imagery and questionnaire measures of general mental imagery use (e.g., IFES, SUIS). A detailed assessment of which imagery processes are affected in bipolar disorder remains to be conducted and should include experimental tasks assessing imagery generation, maintenance, inspection, and transformation (Pearson et al., 2013). It would be intriguing to identify differences between bipolar depression and unipolar depression across such a landscape, in comparison to nonclinical and anxiety control groups. For example, for imagery generation we might predict that relative to a nonclinical control group, bipolar depression will be associated with elevated scores, whereas unipolar depression will have lowered scores.

Third, the current experiment focuses on the impact of a negative event. Future research should test the prediction that bipolar traits will be associated with an increased likelihood for intrusive visual reexperiencing following highly “positive” mania/elation-related events. We have shown that positive films can generate intrusions (Clark, Mackay, & Holmes, 2013; Davies, Malik, Pictet, Blackwell, & Holmes, 2012), but clearer mania/elation film content remains to be developed.

Finally, investigating intrusive imagery in bipolar disorder warrants consideration in a clinical context, regardless of whether it is unique to this disorder or not, but simply because such symptomatology can be distressing and impairing (Holmes, Geddes, et al., 2008). To date research mapping the phenomena has been restricted to suicidal imagery (Hales et al., 2011) and positive imagery (Di Simplicio, Ivins, Close, Goodwin, & Holmes, 2014), whereas anxious imagery has been neglected. Research on manipulating imagery phenomena may also be fruitful. Verbal cognitions are already addressed in cognitive behavioral therapy (CBT) for bipolar disorder, but with limited success (Geddes & Miklowitz, 2013). We hypothesize that imagery-focused CBT (e.g., Arntz, 2012) could reduce negative intrusive imagery after stressors, improve bipolar anxiety, and perhaps aid longer term mood stability.

Arthur Conan Doyle (1888) observed that “where there is no imagination there is no horror.” By using a prospective experimental design, we have been able to shed light on the vulnerability of people with significant hypomanic experience to develop later intrusive images of a stressful event. By inference, compared to others with lower levels of hypomanic experience, stressful events are likely to have a greater impact on this population in daily life. A heightened propensity to engaging in mental imagery may be helpful for the creativity associated with (hypo)mania (Murray & Johnson, 2010) but even be harmful if one is more likely to flash back to the day’s stressors.
